# Outer membrane barrier impairment by envC deletion reduces gut colonization of Crohn’s disease pathobiont Escherichia coli

**DOI:** 10.1099/mic.0.001509

**Published:** 2024-10-15

**Authors:** Tsuyoshi Miki, Masahiro Ito, Takeshi Haneda, Yun-Gi Kim

**Affiliations:** 1 Department of Microbiology, School of Pharmacy, Kitasato University, Tokyo, 108-8641, Japan

**Keywords:** AIEC, bile acids, cell division, Crohn’s disease, gut colonization, outer membrane barrier

## Abstract

Adherent-invasive *Escherichia coli* (AIEC) has been implicated in the aetiology of Crohn’s disease (CD), a chronic inflammatory disorder of the gastrointestinal tract. The presence of *Enterobacteriaceae*, including AIEC, is heightened in the intestines of CD patients. Therefore, inhibiting AIEC colonization in the gastrointestinal tract could be a promising therapeutic intervention for CD. This study aims to assess the potential of EnvC as a novel therapeutic target, examining how disrupting EnvC activity through the deletion of the *envC* gene decreases AIEC gut colonization levels. EnvC serves as a catalyst for peptidoglycan (also called murein) amidases, facilitating bacterial cell division. An AIEC mutant lacking the *envC* gene exhibited impaired cell division. Furthermore, *envC* deletion led to a diminished outer membrane barrier, as seen in our finding that the *envC* mutant became susceptible to vancomycin. Finally, we found that the *envC* mutant is impaired in competitive gut colonization in a dysbiotic mouse model. The colonization defects might be attributable to reduced resistance to colonic bile acids, as evidenced by our finding that increased colonic levels of bile acids inhibited the colonization of the gastrointestinal tract by AIEC strains. The present findings suggest that targeting bacterial cell division through the inhibition of EnvC activity could represent a promising intervention for CD.

## Introduction

Adherent-invasive *Escherichia coli* (AIEC) is isolated more frequently from the intestines of Crohn’s disease (CD) patients compared with healthy controls and is suspected to be associated with this disease [1,3]. Although the aetiology of CD is largely unknown, the microbiome of CD patients is characterized by dysbiosis and a pro-inflammatory state, marked by an excess of *Enterobacteriaceae* [4]. Among these, AIEC strains are particularly recognized as key pathobionts that contribute to the onset and/or exacerbation of CD [5]. AIEC can strongly adhere to and invade epithelial cells in CD patients in a type 1 fimbriae-dependent manner. Subsequently, they persistently colonize the intestinal tract by competing with the microbiota and surviving host-derived antimicrobial responses [5,10]. Furthermore, AIEC can survive and replicate within macrophages [8]. The bacterial cell’s escape from oxidative and envelope stress allows for growth in the phagolysosome microenvironment [11,12]. The survival and replication of AIEC elicit inflammatory cytokines from the infected macrophages [8]. Thus, AIEC can persistently colonize the intestinal tract, which may be linked to CD.

Cell division is essential for bacterial multiplication and is facilitated by a large and dynamic protein complex known as the divisome. In the last stage of cell division, peptidoglycan (also called murein) hydrolases such as Ami proteins and EnvC carry out murein hydrolysis, leading to the separation of daughter cells. Ami proteins are murein amidases that participate in murein hydrolysis by cleaving the amide bond between *N*-acetylmuramic acid and l-alanine [13,14], whereas EnvC stimulates the activity of Ami proteins by interacting with FtsEX. This interaction facilitates conformational changes in EnvC through ATP hydrolysis [15,16]. Thus, EnvC acts as a bridge between effectors (Ami proteins) and the energy source (ATP-bound FtsEX) in cell division. Murein hydrolysis, mediated by the Ami-EnvC-FtsEX system, is ubiquitous in various bacteria, including Gram-negative bacteria [15], Gram-positive bacteria [17,18] and actinobacteria [19,20]. Murein is a pivotal component of bacteria and is absent in human cells. Indeed, for decades, bacterial cell division has been recognized as a potential target for developing new antibacterial agents that inhibit the function of the divisome [21,23].

It has been reported that the Ami-EnvC-FtsEX system is involved not only in cell division but also in the pathogenicity of several bacterial pathogens [24,30]. Notably, *envC* deletions in *E. coli* and *Haemophilus influenzae* alter the protein composition in the outer membrane and periplasm, leading to reduced resistance to antimicrobials such as antimicrobial peptides and serum [26,29]. Similarly, the *Salmonella enterica* serovar Typhimurium ∆*envC* mutant exhibits impairments in the pathogenesis of gastrointestinal infection as a result of reduced resistance activities to bile acids and flagellar motility [27]. Here, we demonstrate that EnvC from AIEC strain LF82 contributes to the colonization of the gastrointestinal tract by this bacterium. We also showed that levels of colonic bile acids act as limiting factors for gut colonization by LF82. We propose that the interplay between EnvC and bile acids influences the outer membrane barrier integrity of LF82. Our findings underscore the potential of EnvC as a promising target for the prevention and treatment of AIEC-related CD.

## Methods

### Bacterial strains and culture conditions

AIEC strain LF82 [1] and its derivatives were employed in this study. Isogenic mutants of LF82 harbouring chromosomal in-frame deletions were constructed utilizing the lambda red recombination system [31] and the template plasmid pKD46-cat, enabling selection in kanamycin-resistant LF82 mutants [9]. The following primers were used for the construction of the ∆*envC* mutant: LF envC-red-FW (5′-GACTGGTAAGCCGCTGTTCATCGTGGAATAATCCCTCCCCGTGTAGGCTGGAGCTGCTTC-3′) and LF envC-red-RV (5′-CGGCAAATGCAAGAACGTTACGACGAAATGGAAACAAAACCATATGAATATCCTCCTTAG-3′). The bacterial strains were cultured in Luria–Bertani (LB) broth at 37 °C with shaking at 160 r.p.m. [10].

### Construction of a complementary plasmid

A complementing plasmid was assembled using DNA fragments containing the *envC* gene, which was amplified by PCR using the following primers: LF envC-*BamH*I-FW (5′-AAAGGATCCGAAGCGCTGGATCACTGC-3′) and LF envC-*Sal*I-RV (5′-AAAGTCGACTTACGACGAAATGGAAAC-3′) from LF82 chromosomal DNA as a template. The resulting PCR products were digested with *BamH*I and *Sal*I and ligated into the corresponding sites of pACYC184, resulting in pACYC-*envC*.

### Bacterial morphology analysis

AIEC strains grown in LB broth were placed on a 1.5% agarose pad, sealed under a glass coverslip and imaged at ×1000 using the Zeiss Axiovert A1 microscope.

### *In vitro* growth analysis of bacterial culture

A growth assay was performed as previously described [9]. In brief, bacteria were grown in the LB medium at 37 °C in a shaker at 160 r.p.m. Optical density at 660 nm was measured to assess bacterial growth.

### Propidium iodide staining

AIEC strains grown in LB broth were mixed with propidium iodide (PI) (Dojindo, Kumamoto, Japan) and further incubated for 5 min at room temperature. The samples were placed on a 1.5% agarose pad and sealed under a glass coverslip. PI-stained AIEC cells were observed and counted at ×1000 using fluorescence microscopy. The attenuation of the outer membrane barrier in an AIEC cell allows PI to enter the bacterial cytoplasm, resulting in the interaction between PI and chromosomal DNA (PI-positive status).

### Determination of MICs for antimicrobials

MICs were determined as previously described [28]. Briefly, AIEC strains were diluted to 1×10^6^ c.f.u. per ml with different concentrations of vancomycin or deoxycholate in sterile LB broth and incubated at 37 °C. After incubation, the OD_595_ was measured using a microplate reader (Bio-Rad Laboratories, California, USA). A positive control was included without antimicrobial agents, whereas AIEC strains were absent in the negative control. MICs were determined as the lowest concentrations of antimicrobials that inhibited bacterial growth by more than 50% compared with the growth of the positive control.

### Invasion assay

HeLa cells were infected with bacteria grown in the LB medium to the late logarithmic growth phase at a multiplicity of infection (MOI) of 10 for 3 h at 37 °C with 5% CO_2_. Extracellular bacteria were then washed away, and 100 µg ml^−1^ gentamicin was added for 1 h to kill any remaining extracellular bacteria. The infected cells were lysed with 1% Triton X-100 for 5 min. The samples were then serially diluted in PBS and spread onto LB agar plates to quantify the cell-invaded bacteria.

### β-Galactosidase assay

Reporter strains (*fimA::lacZ*) were grown overnight in the LB medium and subsequently diluted 1/100 in 5 ml of the same medium and further incubated at 37 °C for 3 h. The β-galactosidase activity was measured as previously described [32].

### Intracellular replication assay

Bacteria grown in the LB medium to the late logarithmic growth phase were added at an MOI of 10 to RAW264.7 cells. The mixture was centrifuged at 1000 ***g*** for 10 min to allow contact between the bacteria and cells. To remove the extracellular bacteria, the cells were then incubated for 10 min at 37 °C with 5% CO_2_, washed twice with PBS and further incubated with 20 µg ml^−1^ gentamicin for 40 min under the same conditions. After removal of the cell culture medium, the cells were lysed with 1% Triton X-100. The samples were enumerated via serial dilution in PBS and plated on LB agar plates to quantify engulfed bacteria. Relative engulfment was calculated at the mean of bacteria recovered at 1 h post-infection relative to the number of inoculated bacteria, set as 1. To quantify the intracellular replicated bacteria, 20 µg ml^−1^ gentamicin was added for an additional period of 23 h 40 min. The relative fold increase (bacterial replication) was expressed at the mean of bacteria recovered at 24 h post-infection relative to the number of bacteria recovered after 1 h of infection, including 40 min of gentamicin treatment, established as 1.

### *In vitro* competitive growth assay

An *in vitro* competitive assay was performed in the LB medium. A 1 : 1 ratio of LF82 or the ∆*envC* overnight cultures was combined, reaching a final concentration of 2×10^6^ c.f.u. ml^−1^ and grown in the LB medium at 37 °C in a shaker at 160 r.p.m. The bacterial culture was subjected to vortexing, and serial dilutions were plated onto LB agar plates containing the appropriate antibiotics to determine bacterial numbers. Both bacterial strains were isolated on agar medium containing ampicillin, while only the mutant strain was segregated using agar medium containing kanamycin. The fold increase was calculated by dividing the number of bacteria incubated for 6 or 9 h by the initial bacterial number (0 h).

### Animals

C57BL/6 mice were bred under specific pathogen-free (SPF) conditions at the animal experimental facilities of the School of Pharmacy, Kitasato University. Alternatively, SPF C57BL/6 mice were purchased from Japan SLC. Mice were housed in our facility to acclimate to the environmental conditions and establish similar microbiota.

### Dysbiotic mouse model of AIEC gut colonization

Mouse infection experiments were performed in mice aged 6–12 weeks as described previously [10]. Briefly, mice were treated with a 2% (wt/vol) dose of DSS (dextran sulfate sodium, molecular mass, 5000 Da; FujiFilm Wako, Japan) in their drinking water for 4 days before infection. Furthermore, the mice were pretreated through oral administration of the broad-spectrum antibiotic streptomycin (25 mg per mouse) and were subsequently challenged orally with 5×10^9^ c.f.u. AIEC strains 24 h later. Alternatively, the mice were orally gavaged with a 1 : 1 mixture of LF82 and the isogenic mutant (total 5×10^9^ c.f.u.). For bacterial quantification, faecal pellets, ileum tissue and colon tissue were collected and homogenized in sterile PBS using a TissueLyser device (Qiagen) for 2 min at a frequency of 25 Hz and then plated at serial dilutions on LB agar plates containing ampicillin or kanamycin. After overnight incubation at 37 °C, c.f.u. were counted. A competitive index was calculated by dividing the population sizes of LF82 by those of its derivative mutants. To regulate the luminal levels of bile acids, the mice were fed a standard rodent chow supplemented with 1.5% colestimide (Mitsubishi Tanabe Pharma), an anion exchange resin.

### Measurement of bile acids in murine faeces

Total bile acids in faeces were quantified as previously described [33]. Briefly, faecal pellets were weighed and then homogenized in ethanol. Bile acids extracted with hot ethanol were evaluated using the Total Bile Acids Test Wako kit (FujiFilm Wako) according to the manufacturer’s instructions.

### Statistical analysis

All statistical analyses were conducted using GraphPad Prism v.10 for MacOS (GraphPad Software). Statistical significance was determined by one-way ANOVA, followed by Dunnett’s multiple comparisons test, the unpaired Student’s t-test, the one-sample t-test or the Mann‒Whitney *U*-test. *P* values below 0.05 were considered statistically significant.

## Results

### LF82 *envC* mutant forms chains of bacterial cells by a failure of cell division

**Fig. 1.**
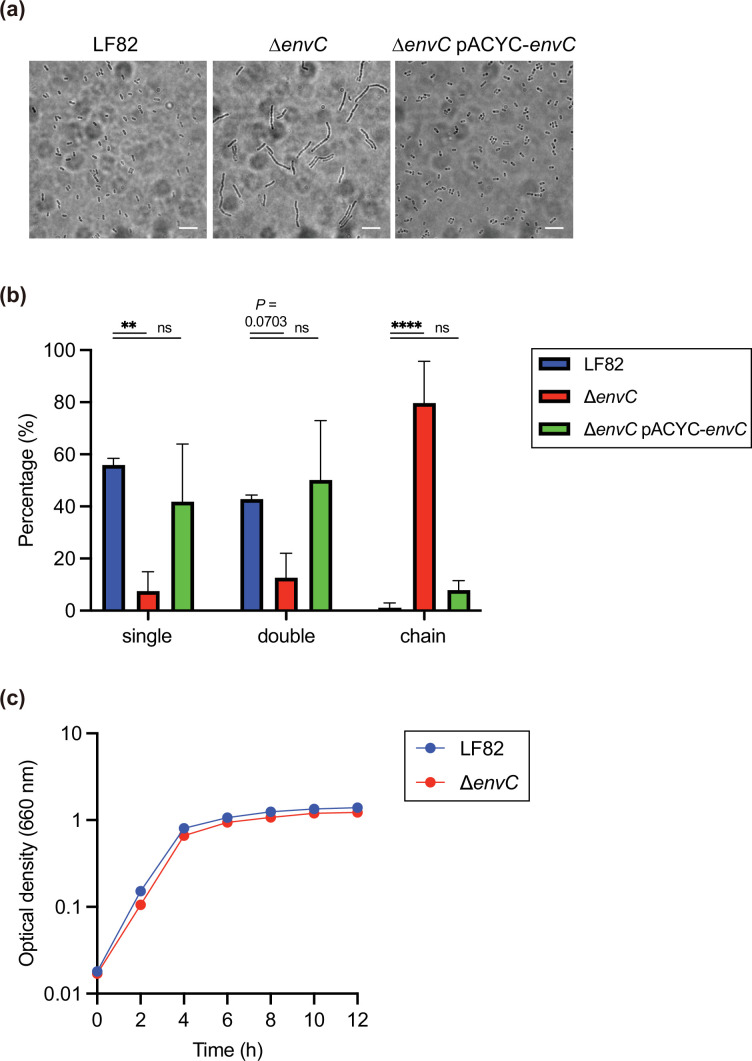
EnvC is involved in cell division. LF82, ∆*envC* or ∆*envC* pACYC-*envC* strains were grown in the LB medium, spotted onto a 1.5% agarose pad, sealed with a glass coverslip and observed using light microscopy. (**a**) Representative microscopy images are presented. Scale bar=5 µm. (**b**) Quantitative analyses of the experiment, with data represented as the means±sd from three independent experiments. ns, not significant; **P < 0.01; ****P < 0.0001; one-way ANOVA followed by Dunnett’s multiple comparisons test. (**c**) *In vitro* growth of LF82 and the ∆*envC* mutant in LB media. Data points represent the means; *n*=3.

EnvC contributes to the cell division process by activating murein amidases of *E. coli* [34]. Therefore, we investigated whether LF82 EnvC is involved in cell division. To this end, we constructed an isogenic *envC* mutant of LF82 by deleting the entire gene and inserting a kanamycin resistance cassette and subsequently performed the cell division test (see Methods). In LF82 grown in the LB medium, almost all of the cells were single or paired (Fig. 1a, b). In contrast, the proportions of single or paired cells of the ∆*envC* were reduced, while those of chains were significantly increased. The introduction of a plasmid encoding LF82 *envC* reversed the cell division defects of the ∆*envC* (Fig. 1a, b). These results indicate that LF82 EnvC is necessary for the cell division process.

We next investigated whether the absence of the *envC* gene could interfere with bacterial growth by measuring the optical density at 660 nm of bacteria grown in the LB medium. The growth rates of LF82 and the ∆*envC* mutant were comparable at all the time points tested (Fig. 1c), indicating that LF82 EnvC is not essential for bacterial growth in a nutrient-rich culture medium.

### LF82 *envC* mutant exhibits an impaired outer membrane barrier

**Fig. 2.**
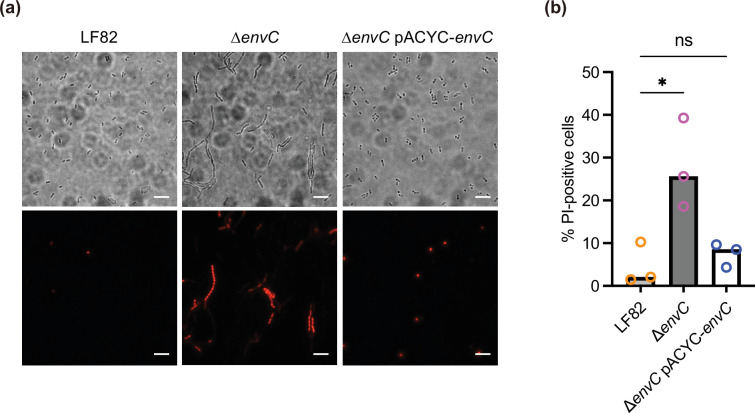
∆*envC* mutant exhibits outer membrane barrier impairment. LF82, ∆*envC* or ∆*envC* pACYC-*envC* strains were grown in the LB medium and subsequently treated with PI solution to evaluate the outer membrane integrity. The bacterial cells were spotted onto a 1.5% agarose pad, sealed with a glass coverslip and observed using light and fluorescence microscopy. (**a**) Representative fluorescence microscopy images are shown. Scale bar=5 µm. (**b**) Microscopy quantification of PI-stained cells. Data are presented as the median from three independent experiments. ns, not significant; ^*^P < 0.05; one-way ANOVA followed by Dunnett’s multiple comparisons test.

Chain formation by *envC* deletion leads to reduced membrane barrier function in *E. coli* and *Salmonella* [26,28]. Thus, to ascertain whether LF82 EnvC could contribute to the establishment of an outer membrane barrier, we assessed the barrier integrity by PI staining (see Methods). A few LF82 cells were PI-positive, whereas the proportion of PI-positive cells was significantly higher in the ∆*envC* compared with that in LF82 (Fig. 2a, b). Reintroduction of a plasmid encoding *envC* restored the PI staining in the ∆*envC*.

Next, we determined the MICs for vancomycin, an antibiotic effective against Gram-positive, but not Gram-negative, bacteria. As expected, LF82 is tolerant to vancomycin due to its presence in the outer membrane (Table 1). In contrast, deletion of the *envC* gene sensitized the cells to this antibiotic, indicating that vancomycin can permeate the outer membrane of the ∆*envC*. These results indicate that *envC* deletion leads to the attenuation of the outer membrane barrier.

**Table 1.** MICs of vancomycin towards AIEC

Strain	Genotype	MICs of vancomycin
LF82	Wild type	>40 µg ml^−1^
T770	*∆envC*	2.5 µg ml^−1^
T830	*∆envC* pACYC-*envC*	>40 µg ml^−1^

Each experiment was repeated three times independently.

### LF82 *envC* mutant has less invasiveness into epithelial cells and is engulfed by phagocytic cells

**Fig. 3.**
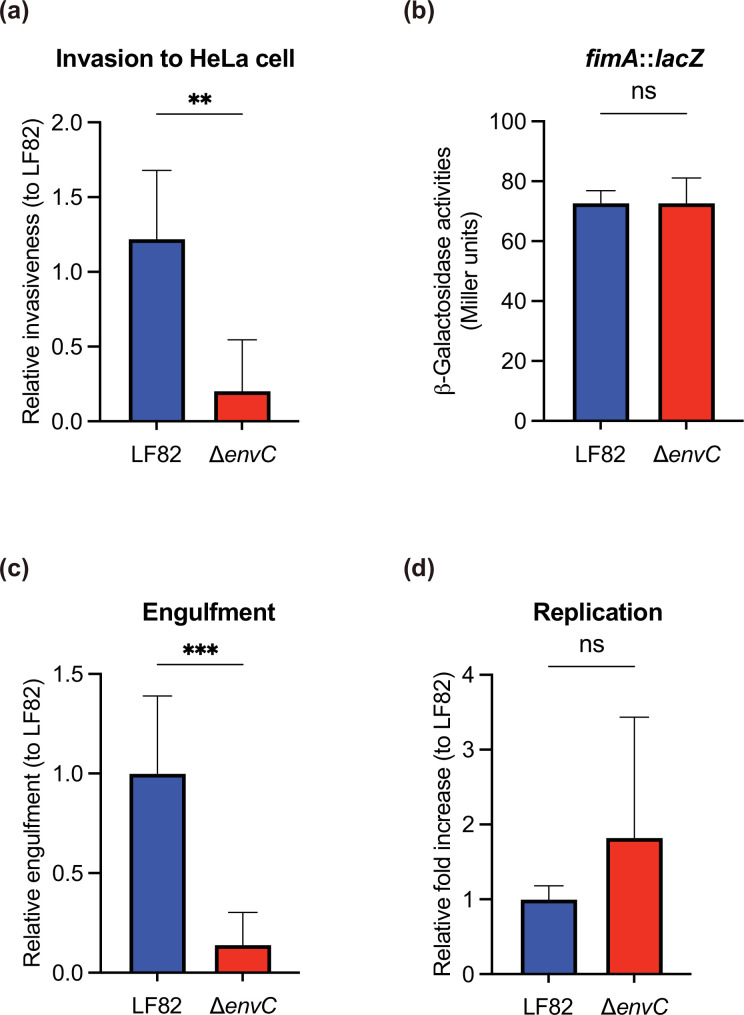
The ∆*envC* mutant has reduced invasiveness into HeLa cells and a decreased engulfment by RAW264.7 cells. (**a**) HeLa cells were infected with LF82 or the ∆*envC* mutant, and their invasion ability was determined by a gentamicin protection assay. Relative invasiveness was compared with that of LF82. (**b**) Transcriptional levels of *fimA* of LF82 and ∆*envC* are presented as units of β-galactosidase activity. (**c and d**) RAW264.7 cells were infected with LF82 or the ∆*envC* mutant, and their capacities for engulfment (**c**) and replication (**d**) with RAW264.7 cells were determined. Relative engulfment and relative fold increase were compared with those of LF82. Each value represents the means±sd from at least three experiments. ns, not significant; **P < 0.01, ***P < 0.001; unpaired Student’s t-test.

The *in vitro* phenotypes of the ∆*envC*, such as cell adherence/invasion and survival/replication, were investigated using HeLa and RAW264.7 cells. The ∆*envC* exhibited drastic impairment of its ability to invade HeLa cells (Fig. 3a). Thus, we then examined whether *envC* expresses the type 1 fimbriae, which are centrally implicated in the adherence and invasion of host epithelial cells. The expression of *fim* operon genes (*fimAICDFGH*) encoding the type 1 fimbriae depends on the *fimA* promoter activity. Experiments using a reporter strain harbouring the *fimA::lacZ* chromosomal fusion showed that the transcriptional activity of *fimA* in ∆*envC* mutant was similar to that in LF82 (Fig. 3b), indicating that the reduced invasion of ∆*envC* into HeLa cells was not linked to the expression of type 1 fimbriae.

Similarly to the invasion into HeLa cells, the reduced phagocytosis capability of the ∆*envC* by RAW264.7 cells was observed compared with LF82 (Fig. 3c). In contrast, no significant disparities were observed in the replication levels of LF82 and the ∆*envC* mutant at 1 or 24 h post-infection with RAW264.7 cells (Fig. 3d). Collectively, our data highlight the role of EnvC in bacterial interaction with host cells, such as epithelial cell invasion and engulfment by macrophages.

### LF82 *envC* mutant is impaired in gut competitive colonization in a dysbiotic mouse model

**Fig. 4.**
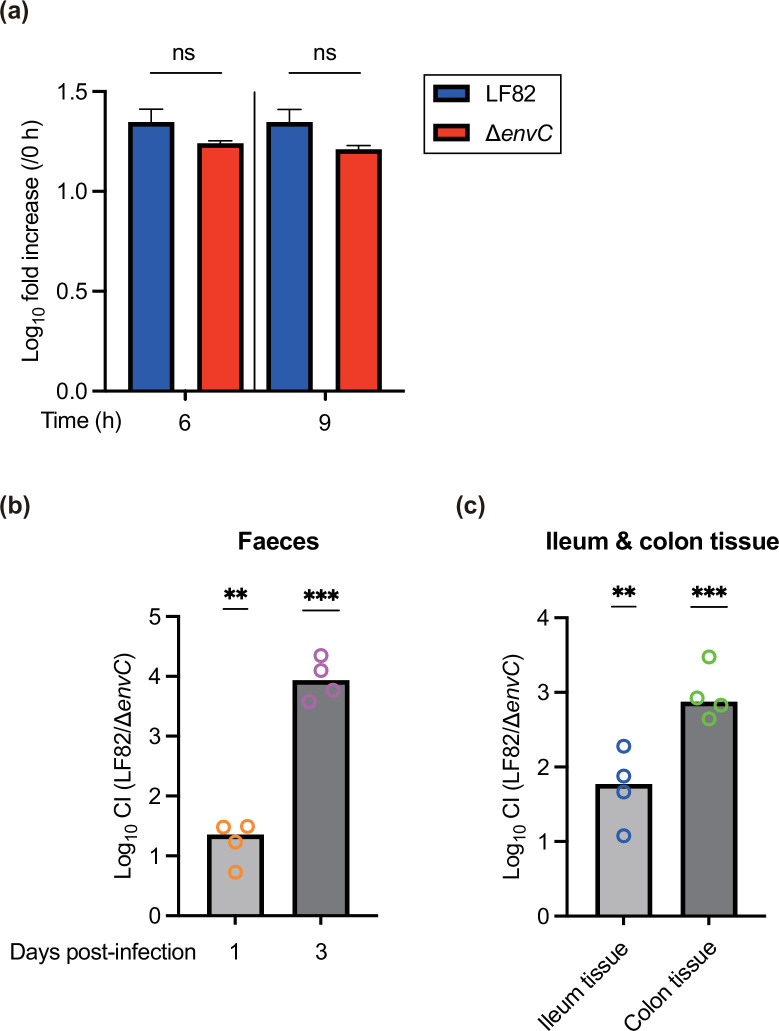
EnvC contributes to competitive gut colonization. (**a**) *In vitro* competitive growth. Fold increase was compared with the bacterial numbers at the starting point (0 h). Bars represent the means±sd. *n*=3. ns, not significant. One-way ANOVA followed by Dunnett’s multiple comparisons test. (**b and c**) DSS-pretreated and streptomycin-pretreated C57BL/6 mice were infected via oral gavage with a 1 : 1 mixture (total, 5×10^9^ c.f.u.) of LF82 and the ∆*envC* mutant. (**a**) Competitive index (CI) values of LF82 strains in faeces on days 1 and 3 post-infection. (**b**) CI values in the ileum and colon tissues on day 3 post-infection. Bars represent the median values. **P < 0.01; ***P < 0.001; one sample t-test.

We investigated whether chain formation by *envC* deletion influences gut colonization in a mouse model. First, we assessed the competitive growth of LF82 and the ∆*envC* by co-culturing both strains in the LB medium. No variations in the replication levels of LF82 and the ∆*envC* mutant were observed at any time points during incubation (Fig. 4a), indicating that each strain grew at a similar rate in a mixed culture. The results of the *in vitro* competitive growth assay also revealed that the chain of the ∆*envC* mutant cells could be sheared into single cells after vortex treatment (see Methods), without affecting colony formation on LB agar plates. Thus, DSS-treated and streptomycin-treated mice were orally gavaged with a 1 : 1 mixture of LF82 and the ∆*envC*. On days 1 and 3 post-infection, the competitive colonization levels of the ∆*envC* in faeces were significantly reduced compared with LF82 (Fig. 4b). Similar results were obtained for the ileum and colon tissues (Fig. 4c), indicating that LF82 EnvC contributes to competitive colonization in the luminal gut of a dysbiotic mouse. Taken together with our previous findings from *in vitro* growth and competitive growth tests, the impaired fitness of the ∆*envC* mutant cannot be ascribed to differences in growth rate and colony formation on agar plates.

### Luminal bile acids limit the gut colonization of LF82

To further explore whether the colonization defects of the ∆*envC* strain are attributable to the reduction of the outer membrane barrier, we focused on luminal bile acids as antimicrobials [35]. To this end, we first measured the MIC values of sodium deoxycholate against LF82, the ∆*envC* strain and the ∆*envC* pACYC-*envC* strain. As expected, the MICs of this antimicrobial towards the ∆*envC* were lower than those against LF82 (Table 2). The complementation strain restored the MIC values. These results indicate that the ∆*envC* is susceptible to bile acids due to the attenuation of the outer membrane barrier.

**Table 2.** MICs of deoxycholate towards AIEC

Strain	Genotype	MICs of deoxycholate
LF82	Wild type	4%
T770	*∆envC*	<0.1%
T830	*∆envC* pACYC-*envC*	4%

Each experiment was repeated three times independently.

**Fig. 5.**
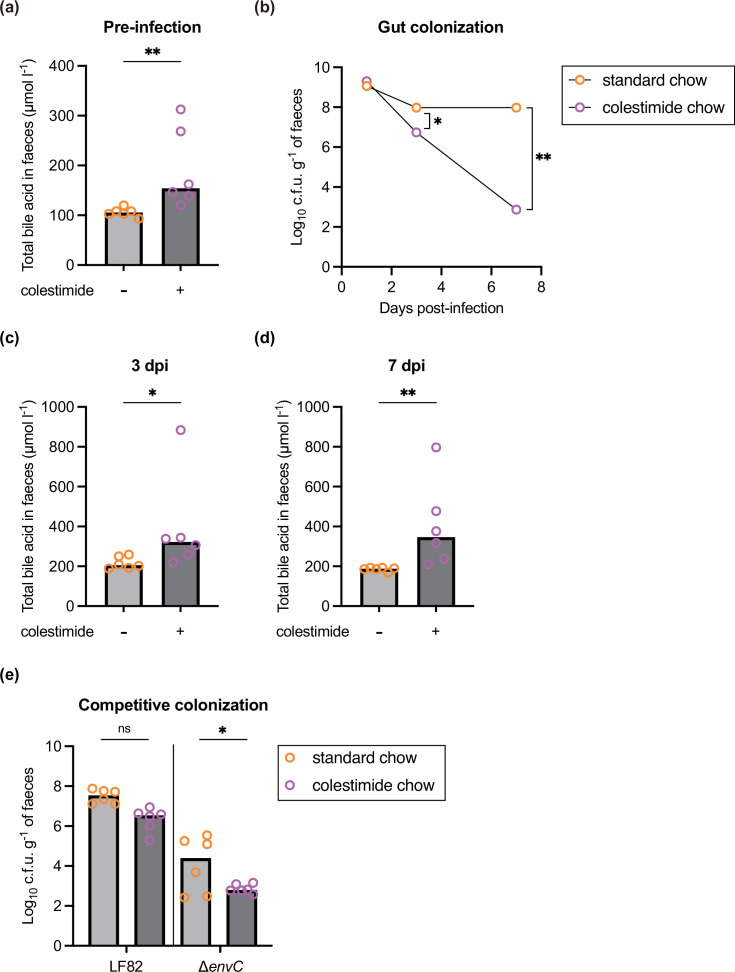
Colonic bile acids foster LF82 clearance. DSS-pretreated and streptomycin-pretreated C57BL/6 mice fed chow containing colestimide resin (colestimide chow) or standard chow (without colestimide) were infected via oral gavage with 5×10^9^ c.f.u. of LF82 or a 1 : 1 mixture (total, 5×10^9^ c.f.u.) of LF82 and the ∆*envC* mutant. (**a**) Concentrations of total bile acids were measured in faeces pre-infection. (**b**) The faecal burden of LF82 was monitored for 7 days post-infection. (**c and d**) Concentrations of total bile acids were measured in faeces on days 3 and 7 post-infection. (**e**) Faecal loads of LF82 and the ∆*envC* mutant on day 3 post-infection. Bars represent the median values. ns, not significant; ^*^P < 0.05; ^**^P < 0.01; Mann–Whitney U test or one-way ANOVA followed by Dunnett’s multiple comparisons test.

We next evaluated whether the antimicrobial effects of bile acids influenced the gut colonization of LF82. To regulate the luminal levels of bile acids, mice were fed chow containing 1.5% colestimide, an agent that binds to and absorbs free bile acids in the gut lumen. The faecal levels of bile acids in mice fed chow containing colestimide were elevated in comparison to those in mice fed control chow (Fig. 5a), indicating that the concentration of luminal bile acids, known for their bactericidal properties against microbes such as LF82, was heightened. Therefore, LF82 was administered via gavage to DSS-treated and streptomycin-treated mice that had been fed chow containing colestimide for 7 days. We observed high levels of gut colonization in mice fed standard chow on day 7 post-infection, whereas LF82 exhibited a significant decrease in colonization of the luminal gut in mice fed chow containing colestimide on days 3 and 7 post-infection (Fig. 5b). Increased levels of luminal bile acids were confirmed in mice fed chow containing colestimide on days 3 and 7 post-infection (Fig. 5c, d).

Next, a 1 : 1 mixture of LF82 and the ∆*envC* strain was administered via gavage to mice fed chow containing colestimide. On day 3 post-infection, the competitive colonization levels of LF82 were similar between the two mouse groups (Fig. 5e). Conversely, the ∆*envC* strain in mice fed chow containing colestimide was significantly impaired in competitive colonization compared with those fed standard chow. Collectively, these results suggest that elevated luminal bile acid levels have the potential to restrict the gut colonization of LF82, likely owing to a direct bactericidal effect. Furthermore, a robust outer membrane barrier enables LF82 to withstand the bactericidal responses of bile acids, leading to persistent colonization.

## Discussion

By contributing to cell division, EnvC plays an important role in bacterial physiology [34,36, 37]. Importantly, given the broad conservation of EnvC and cognate amidase-dependent cell division, the complex could be a promising target for the development of new antibiotics that lyse bacteria [38]. This prospect encourages us to evaluate whether LF82 EnvC could be a therapeutic target for patients with CD. In this study, we demonstrated that the LF82 ∆*envC* mutant is impaired in gut colonization, possibly due to the reduction of the outer membrane barrier. Our results also suggest that luminal bile acids are limiting factors for the gastrointestinal colonization of LF82, which lyse bacterial cells. Taken together, our findings indicate that EnvC inhibitors and the regulation of luminal bile acid levels could hold significance in the therapeutic management of AIEC-associated CD.

Previous studies showed that mutation of an *envC* gene resulted in changes to the protein compositions in the periplasm and outer membrane [26,29]. This could potentially render the bacteria more vulnerable to bactericidal compounds such as serum and antimicrobial peptides. In line with these reports, our data showed that the ∆*envC* mutant of LF82 becomes susceptible to antimicrobials such as vancomycin and bile acids. It is reasonable to hypothesize that the modification of outer membrane protein composition due to an *envC* mutation leads to a compromised membrane barrier in LF82. Future studies will be necessary to elucidate the mechanism by which the ∆*envC* mutant of LF82 becomes susceptible to antimicrobials.

Bile acids are cholesterol-derived molecules that are synthesized in the liver and excreted into the gut lumen to aid in nutrient absorption. In addition, certain bile acids exert antimicrobial effects by targeting membranes and DNA [35]. We here found that the ∆*envC* mutant becomes more susceptible to bile acids than LF82. Similarly, a mutation in the *envC* gene of *Salmonella enterica* serovar Typhimurium leads to hypersensitivity to bile acids [27]. Furthermore, perturbation of Z-ring assembly could potentially make the *Salmonella* cell vulnerable to bile acids [26,39, 40], highlighting a possible link between bacterial cell division and sensitivity to bile acids.

The ileal mucosa of CD patients displays increased adherence and invasion of AIEC into intestinal epithelial cells [6]. Furthermore, the ability to survive and replicate within macrophages represents a virulent phenotype of LF82 [8]. Our data showed that the ∆*envC* mutant exhibits impaired invasion into HeLa cells. A previous study clearly showed that the type 1 fimbriae, encoded by the *fim* operon, enable LF82 to colonize host epithelial surfaces, thereby contributing to cell invasion [6]. As our data indicate, showing identical expression levels of the *fim* operon in the ∆*envC* and LF82, the reduced invasion of the ∆*envC* mutant is not involved in the type 1 fimbriae. Therefore, it is tempting to speculate that the chain formation of the ∆*envC* mutant cells could reduce this mutant’s ability to invade HeLa cells. Similarly, it could be concluded that the chain of the ∆*envC* mutant cells hinders the phagocytosis of LF82 by macrophages, as evidenced by our findings showing that the ∆*envC* was impaired in engulfment by RAW264.7 cells compared with LF82. Importantly, the outcomes of the *in vitro* infection experiments using cell lines could imply that the ∆*envC* mutant cells of LF82 reside predominantly in the gut lumen of the infected host, where bile acids are excreted.

In conclusion, we posit that the development of an EnvC inhibitor could pave the way for therapeutic advancements aimed at alleviating AIEC-related CD. While not directly lethal to bacteria, cell division abnormalities create a significant vulnerability in host colonization, where antimicrobials such as luminal bile acids can effectively eliminate bacteria. Furthermore, unlike antibiotics, an EnvC inhibitor might not exert selective pressure. Hence, EnvC holds promise as a therapeutic target for AIEC-associated CD. On the other hand, since EnvC is a ubiquitous enzyme, suppressing its activity will affect both gut commensals and AIEC. Future work will be needed to clarify whether the EnvC inhibitor provides an effective way to mitigate CD by decreasing levels of *Enterobacteriaceae*, including AIEC.

## Abbreviations

AIEC: adherent-invasive *Escherichia coli*
CD: Crohn’s disease
CI: competitive index
DSS: dextran sulfate sodium
LB: Luria–Bertani
PI: propidium iodide
SPF: specific pathogen-free
